# Global Screening for Human Viral Pathogens

**DOI:** 10.3201/eid0907.030004

**Published:** 2003-07

**Authors:** Norman G. Anderson, John L. Gerin, N. Leigh Anderson

**Affiliations:** * Viral Defense Foundation, Kensington, Maryland, USA; † Georgetown University Medical Center, Rockville, Maryland, USA; ‡ Plasma Proteome Institute, Washington, D.C., USA

**Keywords:** viral pathogens, surveillance, epidemics, shotgun sequencing, biological warfare, biodefense, virus isolation, perspective

## Abstract

We propose a system for continuing surveillance of viral pathogens circulating in large human populations. We base this system on the physical isolation of viruses from large pooled samples of human serum and plasma *(*e.g., discarded specimens from diagnostic laboratories), followed by shotgun sequencing of the resulting genomes. The technology for concentrating virions from 100-L volumes was developed previously at Oak Ridge National Laboratory, and the means for purifying and concentrating virions from volumes in microliters have been developed recently. At the same time, marine virologists have developed efficient methods for concentrating, amplifying, and sequencing complex viral mixtures obtained from the ocean. Given this existing technology base, we believe an integrated, automated, and contained system for surveillance of the human “virome” can be implemented within 1 to 2 years. Such a system could monitor the levels of known viruses in human populations, rapidly detect outbreaks, and systematically discover novel or variant human viruses.

The traditional process of discovering previously unknown human viruses, or variants of known viruses, is neither rapid nor thoroughly systematic. The time between back-calculated initial infection and final identification is often many weeks, months, or even years. For a totally new agent, the estimated interval between initial infection and detailed characterization is variable and depends on the presence of unusual symptoms, the failure to identify a virus after using all available specific tests, the recognition of a unique problem, and, in the past, the ability to grow the agent in culture.

The idiosyncratic nature of virus discovery contrasts with the broad survey approaches characteristic of genomics and proteomics. Only in the relatively small field of ocean viruses has a more inclusive, cataloging approach been tested. Facilitated by the relative ease with which viruses can be isolated from seawater (using commercial filters), investigators in this area have examined a broad and essentially unbiased population of viral agents at the genome sequence level (including phage) and estimated the number of different genomes present (~5,000) (*1*–*3*). One would expect that a comprehensive survey of human viruses, defining what we might term the human “virome” would be, at least conceptually, even more straightforward.

Our proposed approach (Figure), in which large populations are continually monitored for new human-infective viruses, has not been considered technically feasible or medically necessary in the past. For purposes of broad surveillance, we propose using pools of serum or plasma from large numbers of persons, the most likely source of which is excess material collected for routine clinical purposes. These samples would be pooled and processed by using available technology to isolate virus particles en masse, recover viral nucleic acids, produce amplified shotgun libraries, carry out shotgun sequencing of the mixture of viral genomes, and reconstruct these genomes in silico with the techniques originally developed to sequence the entire human genome from random fragments. A central objective is to continually repeat this monitoring process to determine which agents change in abundance over time, find undiscovered agents already present, and detect new viruses when they appear. If successful, we will have for the first time a comprehensive picture of “what is going around.” Surprisingly, most of the systems and technology to carry out this process exist in a basic form and have been successfully employed to survey the extremely varied DNA virus population of the oceans. What remains to be done, to create a system applicable to humans, is primarily its integration, optimization, and implementation in a safely contained environment. We briefly explore the components of this process here and suggest that it can be made operational in less than a year.

**Figure:**
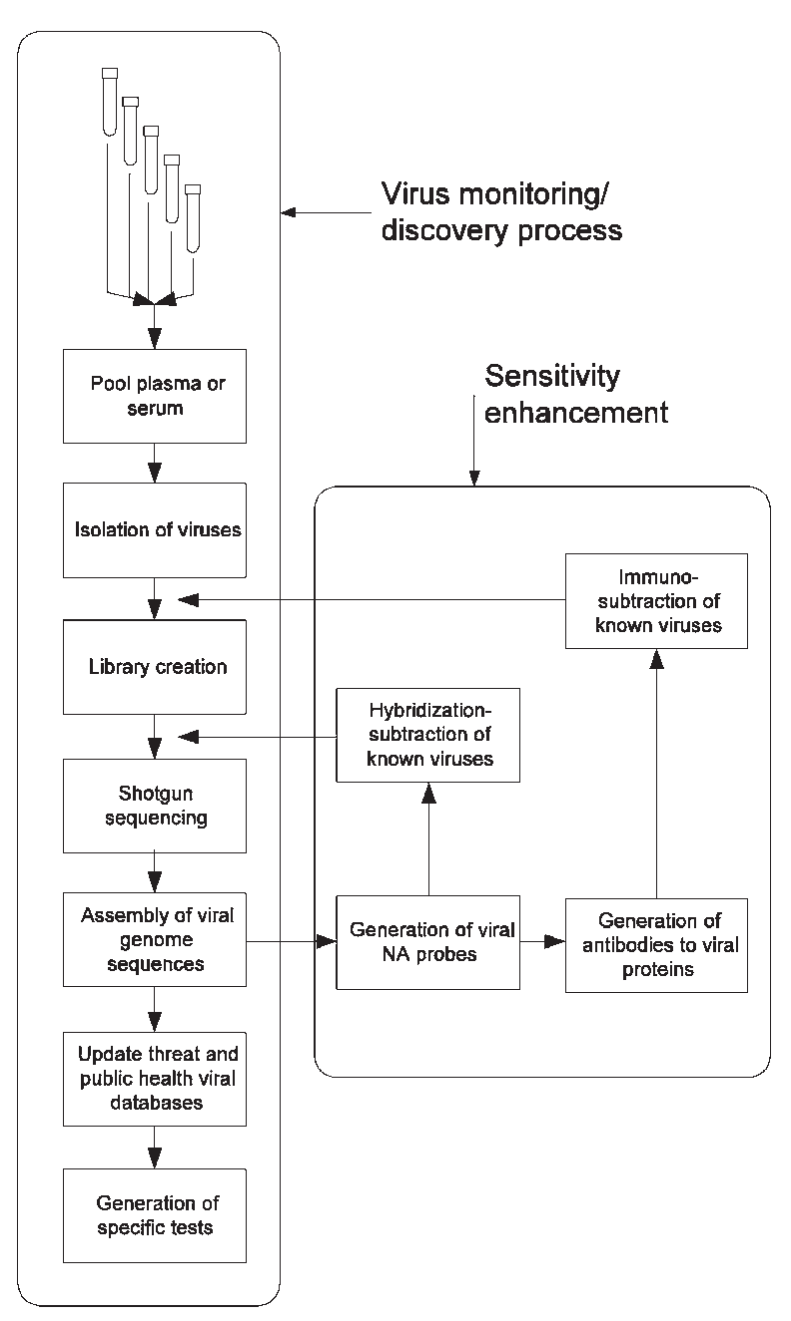
Figure. Schematic representation of a process for systematic discovery of human viruses. The basic process (left vertical series of steps) depends on physical isolation and shotgun sequencing to obtain sequences of frequent and rare viruses. A series of additional steps (right box) can be added to deplete known viruses at two levels, thereby enhancing sensitivity for novel agents.

## Availability of Large Pooled Samples

The major commercial diagnostic laboratories in the United States discard approximately 500 L of excess human serum or plasma each week. This material represents a broad cross-section of patients and illnesses. Plasma viral loads as a function of time after onset of illness are not known for most viral diseases, but they appear to be highest in the initial febrile stages. Since one of the first steps in treating a febrile illness of unknown origin is obtaining a blood sample, we expect that current diagnostic networks contain appreciable quantities of virus. Samples from subpopulations enriched for potential viral illness could also be selected. For those viral diseases in which viremia precedes major illness, the inclusion of large numbers of randomly acquired specimens in the pool (i.e., an unselected pool) offers the best chance of detection. Analysis of pooled samples from a large number of persons should raise minimal privacy concerns.

## Virus Isolation, Sequencing, and Assembly

Methods are required for the routine isolation of all classes of viruses from a pooled sample and for concentrating them by factors of over a million while ensuring that all nonviral nucleic acids have been removed. The concentrates may be dangerously infectious, and sophisticated containment systems will be needed.

The preferred technology for virus concentration from large volumes was developed by the Joint NIH-AEC Zonal Centrifuge Development Project at the Oak Ridge National Laboratory, Oak Ridge, Tennessee, USA, and the Oak Ridge Gaseous Diffusion Plant in the 1960s (*4*). At the outset, researchers wanted to determine whether viruses as a class differed in a systematic way from all other small particles in nature. When the sedimentation coefficients of then-known viruses were plotted against their isopycnic banding densities, nearly all viruses fell into an otherwise essentially vacant area in the center of the plot, surrounded at higher or lower density and higher or lower sedimentation coefficients by various subcellular organelles and macromolecules. This area was termed “the virus window” (*5*). Thus, viruses exhibit a unique size and density range and have banding densities that reflect their combined protein and nucleic acid contents. In addition, viral nucleic acids are shielded from attack by nucleases so that contaminating nucleic acid-containing particles (primarily genomic DNA from apoptotic or disrupted cells) can be selectively destroyed by added nucleases (*6*). The rules of virus isolation are the following: 1) the sedimentation rate (based largely on particle size) falls in a specific range; 2) the banding density in a gradient falls in a specific range; 3) the genome is protected from nuclease attack until the protein (+/- lipid) coat is disrupted; and 4) the major proteins present have sequences that agree with at least part of the genome.

Exploiting the virus window required a two-dimensional separation based on sedimentation rate (S) in one dimension and banding density (rho) in the other, usually carried out in the order: S-ρ. For large-scale isolation and purification, the challenge was to perform these separations continuously and simultaneously in large continuous-flow centrifuge rotors spinning at high speed in a vacuum.

In this scheme, a flowing stream passes inboard of a thin, nonflowing density gradient, held in place against the rotor wall by centrifugal force. The recovered virus forms a narrow band in the gradient, which is recovered after reorienting the gradient to rest at the end of the run. If the flow through two centrifuges is cascaded, the first operating at lower speed than the second, particles having a higher S rate than viruses could be removed from the flowing stream, and the viruses then concentrated and banded in the second higher speed centrifuge, thus providing a large-volume S-ρ separation.

The end result of this work included the design and construction of the K-II large-scale ultracentrifuge (*7*–*10*). This device was designed to recover virus in a high state of purity from 100-L batches of crude influenza vaccine in an 8-hour day (*11*,*12*) and subsequently was used for the large-scale purification of the hepatitis B (Australia) surface antigen (*13*,*14*) from human serum for use as a vaccine and for mass isolation of polyhedral inclusion bodies (*15*). K centrifuges have come into worldwide use for large-scale virus isolation and have been commercially available with little rotor modification for the last 35 years (*16*,*17*). Approximately 200 such systems have been constructed.

Methods are now available to further purify components of complex viral mixtures, to sediment the viruses through gradients containing nonsedimenting zones (e.g., of nucleases), and to concentrate them down to tens of microliters (*18*,*19*) for sequencing or mass spectrometric analysis.

## Ocean Viruses

During the Oak Ridge centrifuge development project, large volumes of test material were required, and seawater, among other sources, was examined as a possible source of virus. The ocean was found to contain examples of almost every known viral form at high titers (*20*), initiating the exploration of marine virology (*1*–*3*,*21*–*30*). Recent data in this field suggest that the oceans of the world contains approximately 10^31^ phage particles of virions (*27*) (c. 22 million metric tons), much of it turning over once per day and including some human pathogens (*28*). This vast mutation engine, even if one assumes a minimal mutation rate, generates the equivalent of hundreds of new complete human genomes per day. That viruses are ubiquitous in the ocean has been demonstrated by studies on samples recovered in widely separated locations from filtration systems installed on surface ships (*29*), nuclear submarines (*3*), and remotely operated vehicles (*30*). Indeed, the entire ocean has an average viral content in the lower range of the viral loads reported for human plasma from viremic patients. Marine virologists have in fact come closest to implementing a surveillance system such as we propose for humans. On limited budgets, these researchers have developed the means of recovering marine viruses from large volumes by filtration (especially well-suited to such a dilute sample), for producing shotgun libraries from them by random amplification (*1*). Marine virologists have also begun to estimate the diversity of marine viruses (*1*–*3*,*27*,*30*) and are reconstructing large numbers of complete viral genomes. In one study (*2*), a 200-L sample of surface seawater was concentrated; ~2 x 10^12^ viral particles were recovered; the DNA was randomly sheared and cloned; and 1,934 fragments were sequenced. Date analysis showed that most of the sequences were from previously unknown viruses. Approximately 3.5% of the total sequence samples overlapped, suggesting that the marine viral community was highly diverse. A unique mathematical analysis (*2*) further suggested that less than 10^4^ different viral types were present and that shotgun sequencing of the most abundant could be done, with existing facilities, in 1 month. Although efforts to date have focused on viruses with DNA genomes, most human viral pathogens have RNA genomes. Genomic sequencing libraries will therefore have to be prepared from mixtures of both single- and double-stranded DNA and RNA viruses (the latter generated by reverse transcription).

The feasibility of the genomics assembly and annotation components of this project derives from the demonstration that the entire human genome could be fragmented, the fragments sequenced, and the original sequence reconstructed from overlaps; that abundant sequencing capacity now exists in search of high-value projects; and that marine virologists have succeeded in parallel ventures. The challenge is to shorten the time of the entire process so useful epidemiologic and viroterrorism response data can be rapidly obtained.

## Remaining Challenges

Recovering viruses from large pools of human serum and plasma and routinely cloning and sequencing the viral nucleic acids (using established shotgun approaches) appears technically feasible. Consequently, the titers of many known human viral pathogens may be estimated routinely, and new viruses (both pathogenic and nonpathogenic) may be discovered systematically.

The initial choice is between filtration and centrifugation. Seawater contains little contaminating material of the size and density of the virus particles, filtration is simple and efficient, and no free nucleic acids have been reported. Plasma and serum present different problems, complicated by the presence of large amounts of protein, some nonviral particles in the virus window, and variable amounts of soluble nonviral nucleic acids (*31*) that must be eliminated. An advantage of centrifugal methods is that all separations, down to banding in microliter gradients, can be (and have been) done with the virions in suspension, a process that avoids aggregation that may occur on filter surfaces.

Several key questions remain to be addressed in a practical project: 1) Can this process be carried out rapidly enough to support a timely therapeutic or prophylactic response to a new agent (natural or engineered)? 2) Will the novel virions that originated from one or a very few infected persons be recovered and detected? 3) Can the affected persons be located?

## Speed of Operation

Samples can be collected weekly, and materials rapidly transported to one or more processing sites. Virus isolation, library construction, and preparation of clones for sequencing require <7 days but the time may be compressed into <4 days with a 24-hour per day operation. Sequencing time will be determined by available capacity, but since capacity is abundant, with large increases in sight, extensive library sequencing (e.g., 10–100 megabases) could be carried out in 2 to 3 days. Less than 1 day would be required to assemble viral genomes as contigs in silico. By the conduction of each step in sequence, turnaround (serum pool to raw sequence data) could be completed in approximately 10 operating days. Additional time would be required for bioinformatics analysis of the data and annotation, but prevalence and novelty conclusions should be available almost immediately. These estimates assume an integrated and fully developed system in continuous operation, analogous in some respects to those monitoring computer viruses.

## Sensitivity

The mass of virus ultimately recovered from pooled human plasma is difficult to estimate in advance. If the average virus has a mass of 1.0 x 10^-15^ g; if the average titer of an infected person is 10^6^ virions/mL, and if 0.1% of the samples are from viremic patients, then ~0.5 μg of virus would be recovered from each 500-L pool, substantially more than the few nanograms required to make a large library with current technology (*1*,*2*). If the average sample contributed to the pool was 1 mL, and if the final concentrated virus were in 1 mL, the final concentration of a totally new virus would be close to that in the original individual sample. The possibility of detecting all viruses for which polymerase chain reactions (PCR) primers are available, down to contributions from single patients, therefore exists.

## Dynamic Range

T The problem of dynamic range can be addressed in three ways. First, given large sequencing capacity, one could sequence deeply into the libraries (millions of clones instead of a few thousand), thereby detecting parts-per-million sequences. Second, one could apply antibody-based affinity methods to deplete known viral particles from the initial concentrated viral sample. Third, one could use subtractive hybridization to remove known viral genomic sequences to further enrich libraries in novel genomes. The last two approaches can be progressively extended as viruses are characterized to provide a continuous increase in sensitivity to new agents (Figure).

## Identification of Viral Sources

Two approaches can be used, if necessary, to link viruses to patients. In the first approach, viruses would be tracked geographically, first in terms of large regions, and then, sequentially, in terms of smaller areas. Detecting a new agent in large pooled samples would thus be repeated in smaller, localized pools that had been combined hierarchically to generate the larger pool (*32*).

A potentially more efficient approach involves overlapping subpools designed such that a new viral sequence can be assayed (e.g., by PCR) in the subpools and the affected persons identified in one step. To achieve this result, each sample is added to a series of different pools, the identity of these subpools providing an “address” of the sample (*33*). This process can be visualized by analogy to a 3-D chessboard, where each position represents a sample, and the subpools are the various planes parallel to the top, front, and side: each sample would contribute to three subpools. In practice, additional pools would be created to provide a relatively unambiguous means of backtracking from the pattern of subpools positive for a specific sequence to one or a few persons.

## Viral Pathogens That May Be Missed

Not all human viral pathogens will be detected easily by analyzing plasma or serum samples. Neurotropic viruses such as rabies, for example, are found in cells and tissues and do not appear free in serum or plasma in appreciable amounts. Thus, these viruses would escape the screening system described to this point. Although using rabies for viroterrorism would be unlikely, such viruses are of great public health interest, and efforts should ultimately be made to include them in any global screening system.

The rapid turnover of viruses found in plasma suggests that they are removed into cells, and that appears to be generally true. Centrifugal S-ρ technology was originally developed for cell fractionation with the aim of isolating viruses from tumors, cells, and tissues (*5*). Trace quantities of virus could be added to tissue homogenates and recovered in a high state of purity. The basic technology therefore exists for isolating viruses from lymphocytes and a variety of different tissues. At a later stage, the proposed approach should be applied to whole blood (with cells lysed before virus recovery), nasal washings, tissues, and other potentially virus-laden samples.

## Automation and Containment

To routinely detect new and potentially lethal viruses, researchers may need to create completely automated and contained laboratories that continually search for and sequence viruses from a wide variety of sources to hone skills; demonstrate efficiency; and develop improved systems, methods, and reagents.

Containment was of great concern in the original Manhattan Project to deal with radiologic hazards, and in the Oak Ridge centrifuge project (*34*) to contain infectious agents. Containment systems have since evolved in two directions. In biological sciences, interest has centered on schemes to allow investigators to work in a safe environment using essentially the same tools they would use on an open bench. As a result, designs has evolved in which human operators are contained in “space suits.” In nuclear programs, in contrast, (where containment systems actually originated), operators are completely isolated from the contents of “hot cells,” and operations in these cells are done remotely with specially designed equipment. Given the national urgency for automated systems for virologic studies, completely robotic automated systems should be developed, analogous to those used in nuclear research, because 1) the concentrated samples to be analyzed are potentially extremely dangerous, 2) work must be done without interruption, and 3) speed and precision depend on automation. Although the K-II centrifuge has not thus far been automated for totally remote operation, including cleaning between runs, this is not an overwhelming problem and should involve cascaded centrifuges, as was done for the mass isolation of Tussock moth polyhedral inclusion bodies (*15*).

## False Positives, False Negatives, and the Price of Errors

All current diagnostic tests have the potential for false-negative and false-positive results. In the atmosphere of an actual terrorist attack with a biological agent, however, the consequences of these false outcomes place an enormous strain on public services, as demonstrated by the recent anthrax episodes. A false positive triggers highly disruptive responses, whereas a false-negative result exposes the population to the obvious health concern. The approach described here reduces the possibility of such outcomes and only assumes that the virus has the expected biophysical properties of size and mass and an internalized genome. If a particle with the appropriate biophysical properties coincides with an internal genome that codes for structural proteins that are also found in the same fraction, the possible number of false-positive error is acceptably small and few mechanisms exist by which false evidence for a truly nonexistent viral sequence might emerge from the process described. The level of false negatives depends not only on the overall quality of the analysis but also on its sensitivity to rare events, i.e., the dynamic range. For sequence-based analyses, sensitivity depends on the frequency with which a sequence appears in a fragment library, the number of clones produced, the efficiency of known virus subtraction (if applied), and the number of different clones sequenced.

The number of intentional false positives (duping) is another matter and one that has two aspects. First, intentional introduction of unexpected pathogens or their genes into a global analytical system is itself a terrorist act and one that should be detected and known. To insert a substantial amount of recoverable viral particles into the sample collection system, a person trying to deceive the system would have to engineer and grow these viruses—an act sufficiently close to actual bioterrorist use that it requires detection, whether the agent is a serious human pathogen or not. The second aspect concerns the best response to the suspicion that such duping has occurred. Complete sequencing of the agent(s) involved would be important since one cannot initially distinguish a genuine sample from one used in duping. Only after extensive further studies and the demonstration that an outbreak has not occurred may the sample be determined not to be of patient origin.

Forewarned is forearmed. Given advance notice, even by weeks, of an impending viral outbreak, the hope exists that the tools and imaginations of molecular biology will find the means to prepare some effective biological defense.

## Medical Contributions of Global Surveillance

The problem of developing new antiviral agents, especially those specific for one or only a few viral diseases, is circular. Without such treatments rapid agent identification is not necessary, but without such identification no pressing commercial justification for developing specific antiviral agents exists (except for HIV) because they will not be widely used. To be successful, diagnosis and therapy must be linked. This project would assist in forging that link.

## Conclusion

Isolating and sequencing the genomes of a wide variety of viruses from pools of the excess human serum and plasma currently collected and discarded by large diagnostic laboratories is now technically feasible. This collection and analysis process could allow new or unknown pathogens to be identified in the first, or at most second, round of infection. Not all human viral pathogens will be present in such mixtures, but they will include a large fraction of all known highly infectious viral agents. Since the core technologies, though varied, are highly developed, we believe that the initial feasibility studies could be completed in 1 year.

Former Senator Sam Nunn and William H. Wulf, president of the National Academy of Engineering, have both proposed setting up a project concerned with bioterrorism, modeled after the Manhattan Project. We believe that the project described could form the nucleus of such an effort and suggest that lessons learned in the Oak Ridge centrifuge project may apply. As noted by Alvin Weinberg, that project was the first (and hopefully not the last) large-scale project in the biological sciences in which facile access to a wide range of technologies was provided, on the model of the original Manhattan Project (*35*).

In separate articles, we will discuss the possibility of linking rapid detection to rapid responses, including vaccine and therapeutic antibody development, in an attempt to abort epidemics caused by new viruses while they are in progress.
